# Synchronous Upper Squamous and Lower Adenocarcinoma of the Oesophagus: A Rarely Reported Case Treated with Palliative Chemotherapy and Stenting

**DOI:** 10.1155/2017/2713589

**Published:** 2017-07-31

**Authors:** Niall Hardy, Justin Kelly, John Conneely, Barry Kelleher

**Affiliations:** ^1^Department of Hepatobiliary Surgery, Mater Misericordiae University Hospital, Dublin, Ireland; ^2^Department of Gastroenterology, Mater Misericordiae University Hospital, Dublin, Ireland

## Abstract

Oesophageal cancer is divided into two main subtypes, squamous and adenocarcinoma. It is the eighth most common cancer in the world with squamous more common in the developing world and adenocarcinoma most prevalent in the developed world. Incidences of concomitant squamous carcinoma with adenocarcinoma are exceedingly rare with only a few documented occurrences in the form of case reports existing. Here we report a case of synchronous squamous and adenocarcinoma of the oesophagus occurring in an 81-year-old lady with dysphagia, weight loss, and no identifiable risk factors.

## 1. Case Report

An 81-year-old lady presented for further evaluation of progressive dysphagia with associated weight loss. The patient reported a 4-month history of unintentional weight loss of 5 kg. She is a life-long nonsmoker with no past medical history other than hypertension and hypercholesterolaemia, for which she takes a statin and an amlodipine/valsartan combination. Bloods were all within normal limits except for a mildly elevated creatinine at 105 umol/l (Hb 13.5 g/dl, MCV 87.9 fl). Physical exam was noncontributory. In particular, there was no evidence of secondary metastatic disease.

**Figure 1:**
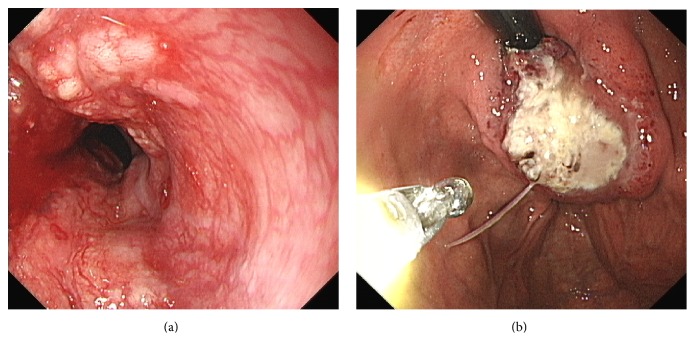


An oesophagogastroduodenoscopy (OGD) was carried out which was significant for two distinct oesophageal tumours. The first was found at 30–35 cms with an appearance of bulging mucosa (Figure 1(a)). A second tight stricture was seen at 40–46 cm abutting the oesophagogastric junction with extensive mass lesion extending into the fundus of the stomach (Figure 1(b)). The intervening oesophagus from 35 to 40 cm was normal in appearance as was the remainder of the OGD with the scope passing without difficulty to the second part of the duodenum. There was no endoscopic evidence of Barrett's oesophagus.

Multiple biopsies were taken and a staging CT of thorax, abdomen, and pelvis was performed. This confirmed the two separate tumours along with celiac adenopathy suggesting locoregional spread.

**Figure 2:**
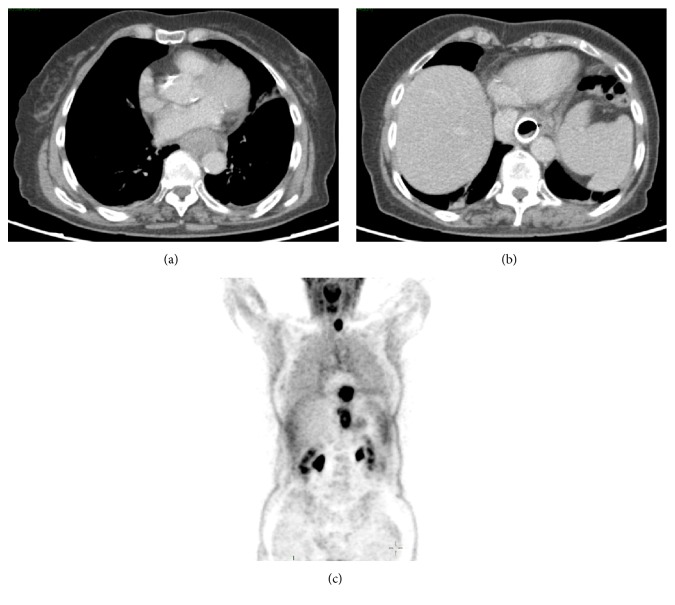
Axial contrast enhanced CT at the level of the mid-oesophagus (a) demonstrates a circumferential soft tissue oesophageal mass with increased metabolic activity (SUVmax 15.0) on the PET component of the study (c). Axial contrast enhanced CT at the level of the gastroesophageal junction (b) demonstrates soft tissue thickening surrounding the gastric stent in situ. The PET component of the study (c) at this level shows increased FDG avidity (SUVmax 14.2) consistent with a second tumour. Incidental note of a FDG avid left thyroid nodule.

PET/CT showed that diffuse metabolic activity to a maximum of 14.2 SUV was evident at both tumour sites and there was associated gastric wall thickening compatible with extensive local spread of the distal mass. Large metabolically active lymph nodes at the level of the celiac axis measuring up to 15 mm were evident with a maximum SUV of 4.5 (Figure 2).

**Figure 3:**
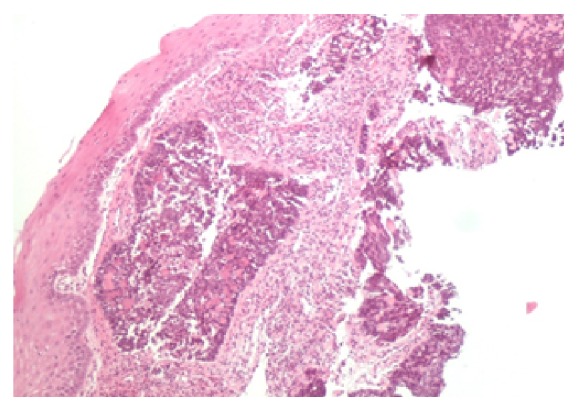
Invasive high grade squamous cell carcinoma.

**Figure 4:**
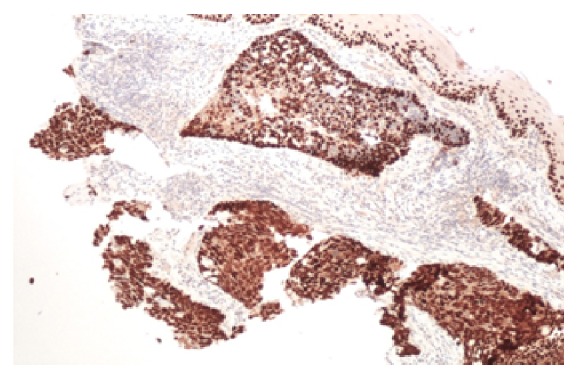
SCC positive for P63, CK 5/6, and CAM5.2.

Analysis from the biopsies confirmed the presence of two histologically distinct masses. Specimen A from the mid-oesophagus showed high grade carcinoma with some features suggesting neuroendocrine differentiation (Figure 3). Overlying squamous mucosa shows moderate to severe dysplasia. Immunohistochemistry showed positive staining for CK 5/6, P63, and CAM 5.2 confirming a diagnosis of SCC (Figure 4). No histological evidence of Barrett's oesophagus was seen.

**Figure 5:**
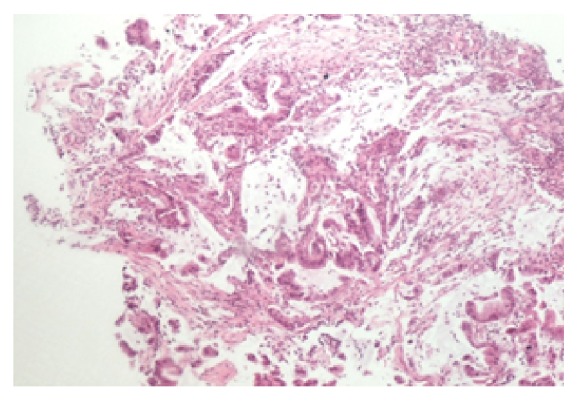
Invasive moderately differentiated mucinous adenocarcinoma.

Specimen B from the lower oesophagus showed invasive moderately differentiated mucinous adenocarcinoma which was HER 2 negative (Figure 5).

**Figure 6:**
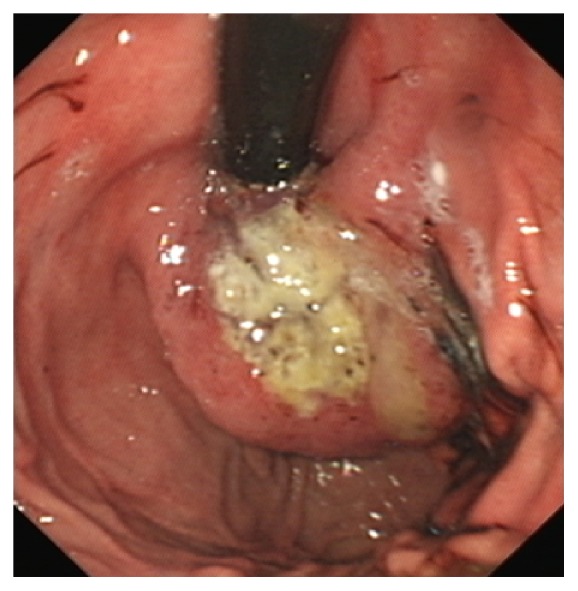
Lower oesophageal mass prior to stenting.

**Figure 7:**
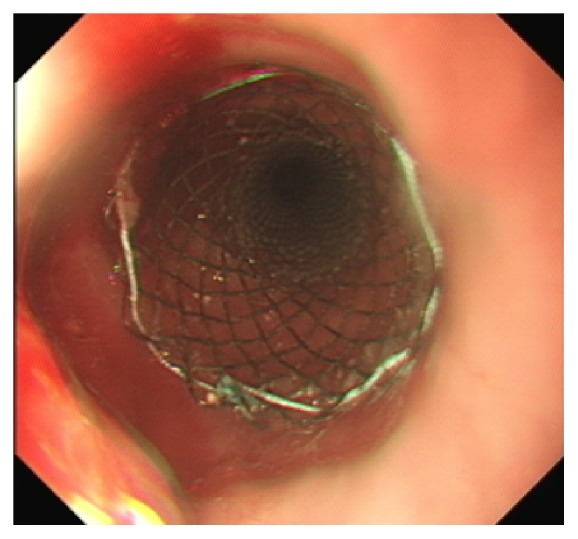
Lower oesophagus poststenting.

Progressive dysphagia warranted a repeat OGD with the insertion of an oesophageal stent.  Figure 6 demonstrates the lower oesophageal mass prior to stenting. A 12 cm partially covered Wallflex stent was placed across the lower malignancy (Figure 7). The second stricture was not stented as it was felt that covering both strictures with one stent would offer a suboptimal result.

Her case was subsequently discussed at our hospital MDT. It was decided that radiation therapy was unsuitable in this case. A radiation field of at least 20 cms would be required to encompass both proximal and distal tumours along with the positive lymph nodes. Safe delivery of full dose radiation to this extent in combination with chemotherapy would not be feasible.

Chemotherapy was commenced as per the standard CROSS regime of weekly paclitaxel and carboplatin as it was felt that these agents would treat both cancer types. This was administered for twelve weeks after which time a restaging CT scan was performed. This CT showed a slight increase in size of one of the previously noted celiac lymph nodes. As a result, the chemotherapy regime was switched to bolus fluorouracil (5 FU) and folinic acid. This was continued for three cycles. This treatment yielded a good outcome with the patient surviving for 15 months from initiation of treatment.

## 2. Discussion

We present a case report of synchronous upper squamous and lower adenocarcinoma of the oesophagus in an 81-year-old female. Although cases of synchronous oesophageal and gastric cancers are becoming more commonly reported, cases such as this remain exceedingly rare in the literature [1, 2].

Risk factors for each of these malignancies differ with gastroesophageal reflux disease and obesity predisposing to adenocarcinoma, compared to alcohol and cigarette smoking being the most significant risk factors for squamous cell carcinoma [3]. None of these risk factors were present in our case. Furthermore, adenocarcinomas of the oesophagus are three to four times more common in males than females [3].

The presence of synchronous tumours of the oesophagus presents a therapeutic challenge. No guidelines currently exist for their management and the only published experience is in the form of case reports.

Treatment modalities include surgery, chemotherapy, and radiation therapy alone or in combination. Chemoradiotherapy has been shown to definitively treat squamous cell carcinoma of the oesophagus even in elderly patients. The presence of a synchronous lower adenocarcinoma however precluded the use of radiotherapy in this instance as the field of radiation required would be too extensive.

Similar cases to this in the literature are scarce. Geramizadeh et al. report a case of synchronous oesophageal malignancies; however, the squamous component was only discovered after oesophagectomy for, what was thought to be, a solitary adenocarcinoma. This patient died in the postoperative period from bronchus rupture and pneumothorax [1].

Maleki et al. report a comparable case in a 58-year-old Iranian woman where chemotherapy and radiotherapy were used and tumour progression was delayed for 19 months from initiation of therapy [2].

A case of resectable synchronous oesophageal disease has not yet been published in the literature. Kano recently reported the successful treatment of synchronous gastric and oesophageal cancers with the multimodal approach of chemoradiotherapy followed by gastrectomy [4].

In this case, our patient was not a suitable candidate for surgery; therefore, palliative chemotherapy and stenting were offered. Taxol and carboplatin agents have been shown to treat both squamous and adenocarcinoma of the oesophagus with acceptable side effects [5]. The dysphagia, which was having a significant impact on quality of life for this patient, was addressed with the use of an oesophageal stent. Stents have been shown to successfully alleviate dysphagia and improve the nutritional status of patients through improving oral intake [6].

We herein describe the use of palliative chemotherapy and oesophageal stenting in synchronous oesophageal tumours to both delay tumour progression and provide symptomatic improvement in an 81 year old. This is only the second case report we could identify in which a similar treatment strategy was used and provides more evidence that this approach can be successfully employed for short to medium term survival in these extremely rarely encountered patients.
